# Ethological and Physiological Parameters Assessment in Donkeys Used in Animal Assisted Interventions

**DOI:** 10.3390/ani10101867

**Published:** 2020-10-13

**Authors:** Michele Panzera, Daniela Alberghina, Alessandra Statelli

**Affiliations:** 1 Centro Universitario Specializzato per gli Interventi Assistiti con gli Animali, Università degli Studi di Messina, 98168 Messina, Italy; mpanzera@unime.it; 2 Dipartimento di Scienze Veterinarie, Università degli Studi di Messina, 98168 Messina, Italy; daniela.alberghina@unime.it

**Keywords:** animal welfare, human-animal interactions, donkeys, AAIs

## Abstract

**Simple Summary:**

The study aimed to obtain scientific data useful for the development of methodologies and standardized protocols for welfare donkey monitoring during animal-assisted interventions (AAIs). For this purpose, on thirteen donkeys, ethological tests and physiological parameters (heart rate (HR), heart rate variability (HRV), and root mean square of successive differences (rMMSD)) were assessed during AAI sessions. The AAI sessions determined modifications of tested parameters. These preliminary results can be considered as a starting point for the investigation of the welfare in donkeys used in AAIs.

**Abstract:**

Background: Few studies have been performed to identify objective indicators for the selection of therapeutic donkeys or to assess their welfare during animal-assisted interventions (AAIs) Objective: This study aimed to evaluate the response to the ethological test and the modifications of physiological parameters in donkeys subjected to AAI sessions. Methods: Thirteen donkeys were subjected to a behavioral evaluation during an AAI session. Heart rate, heart rate variability, and root mean square of successive difference values were detected. Results: Statistically significant changes in the tested parameters were observed during AAI sessions. Conclusions: In donkeys, there was a neurovegetative involvement during AAI sessions. Our data give a contribution to the evaluation of donkey welfare during AAIs.

## 1. Introduction

In the area of social and health interventions, animal-assisted interventions (AAIs) can be useful from a therapeutic and educational point of view to improve the living conditions of patients affected by various degrees of neurological and mental disabilities, as a part of the treatment plan. In particular, in children with an autism spectrum disorder, an improvement in social functioning and interaction, with reductions in stress, anxiety, and loneliness, has been observed. Meanwhile, in subjects affected by multiple sclerosis, spinal cord injury, and stroke, a decreased spasticity with improved balance has been recorded. In addition, in adolescents affected by mental health, an increase in engagement and socialization behavior, and a reduction in disruptive behavior has been observed [1,2,3,4,5]. In AAIs with the help of horses, the postural and kinematic types of physical benefits are predominant [6,7,8,9,10,11,12,13,14,15,16]. In AAIs with the help of donkeys, the benefits regarding mental health are predominant—in fact, donkeys are more indicated in the therapeutic approaches towards emotions and communicative functions and mental retardation [2,5,17].

Nevertheless, in the United States and most European countries, AAIs have begun to be implemented in an objective way [18,19,20]; in particular, in Italy methodologies for the evaluation of animal’s eligibility and welfare during AAIs has been published [18,19,20,21].

The scientific interest in AAIs is concentrated upon the identification, quantification, and validation of ethological and physiological parameters [22,23] for the evaluation of animal welfare [24]. This aspect represents the frontier of ethological research applied to the monitoring of animals used for the multiple forms of AAIs: animal-assisted activity (AAA), animal-assisted education (AAE), animal-assisted therapy (AAT) [25,26,27]. In multiple human nosographic frameworks, AAIs have been examined to demonstrate their beneficial effects [2,6,7,8,28]. Regarding animals used for AAIs, the modification in ethological and physiological parameters observed are not homogeneous and are not often supported by validated methodologies [9].

Studies on the use of horses for the AAIs were directed to establish the suitability of the horses rather than the effects on horses’ welfare. For example, a standardized model for the management (housing and training) of horses specifically used for AAT has been formulated [29]. Biometric parameters, gait quality, and temperament were reported to be important aspects of the selection of horses for equestrian rehabilitation programs [30,31,32,33]. In donkeys, as observed in horses [24,34], the respect of particular ethological components (maintenance behavior, social space, countability, escape distance, family group) is fundamental in the management of donkeys themselves. Moreover, the application of knowledge on the ethogram of *Equus caballus L*. is not always reflected in that of *Equus asinus L.* [35,36,37,38,39].

Biomechanical and kinematic characteristics of the horse’s gait promote the improvement of psychomotor skills, whereas donkeys do not promote benefits in the kinematic aspects. Thanks to their greater empathic sensitivity, donkeys have the potential to improve socialization behaviors and mental health. The more general ethological knowledge on the influence of management conditions on the quality of the animal’s life allows us to believe that animals used for AAIs should express their normal behavior. In particular, as a social and gregarious species, they should express their empathic abilities in the care relationship [40,41].

In AAIs, the evaluation of the empathic abilities of animals is based on the measurement of their emotional responses. ln different experimental contexts and mainly concern, changes in the level of arousal—mediated through the autonomic nervous system (ANS), the hypothalamic–pituitary axis and the adrenal gland (HPA)—can be monitored through adaptive physiological responses to heart rate (HR), blood pressure (BP), breath frequency (BF), pupil diameter, sweat, corticosteroid levels and neurochemicals [42]. These neurovegetative responses are similar to those observed in humans [43,44]. Through the relationship between ANS physiological profiles and the ethological indicators of emotional experience, it is possible to arrive at the evaluation of animal emotions. In humans, the meta-analysis of 22 ANS parameters showed that changes in cardiovascular parameters would seem to differentiate between positive and negative affective-emotional components of emotions [45]. Other studies have shown that 11 ANS parameters (cardiovascular, electrodermal, and respiratory) differ (with an accuracy of 85%) between fear, sadness, and neutral emotional responses [46].

In animals, the research into neuroethology and cognitive ethology concerns the possibility of distinguishing between positive and negative emotions (valence), together with measures of intensity (excitation). In horses, for example, the generic behavioral response of “stress” formed the basis for measuring how they “feel” the modalities of management and training [47,48].

The paradigm of the novel object test is considered highly stressful, with a consequent surprise effect, short-term responses to fear, and avoidance, together with a significant increase in HR and a decrease in heart rate variability (HRV) [24,49]. A positive correlation was found between behavioral signs of anxiety and increased HR in isolated horses [50], whereas Peters et al. [51] found significant increases in HR in horses during food expectation with unchanged HRV values.

HR is considered as a measure of stress in animals because it reflects the ratio between the vagal (which reduces HR) and the sympathetic tone of ANS (which increases HR) [52,53,54,55]. At rest, vagal regulation dominates, but the influence of the sympathetic components of ANS increases with physical activity. HR values in the resting horse are 28–40 beats/minute (bpm), although it can change with age, breed, body weight, and associated problems [56]. The analysis of HRV can be applied together with HR, in the evaluation of stress, including the assessment of mental stress response in horses [22]. HRV is a variation of the R-R interval of two adjacent QRS complexes of electrocardiogram.

This change can be studied with time-domain analysis or frequency-domain analysis. Time-domain analysis is adopted to quantify changes in R-R intervals (in milliseconds) over time through the assessment of the average R-R interval, the standard deviation of all R-R intervals (SDNNs), and the square root of the sum of the square of differences between successive R-Rs (root Mean Square of Successive Differences—rMSSD). The rMSSD is the time domain parameter that measures the high frequency of beat-to-beat variations that stand for the vagal regulatory activity [23].

The heterogeneous terminology of therapeutic activity descriptions and the lack of standardized protocols of AATs and AAEs suggest the need to adopt validated methodologies and tools for the objective evaluation of the changes induced by the care relationship with equids.

The aim of the study was monitoring ethological and physiological parameters which could be useful for the future development of methodologies and contributing to the development of a standardized protocol for welfare donkey monitoring during AAIs.

## 2. Materials and Methods 

### 2.1. Compilation of Control Group (CG) and Experimental Groups (EG_1_ - EG_2_) Ethograms 

All procedures were performed in full accordance with Italian legal regulations (National Directive n. 26/14 –Directive 2010/63/UE) and the guidelines for the treatment of animals in behavioral research and teaching of the Association for the Study of Animal Behavior (ASAB).

Prior to the acquisition of the ethological data, all animals were subjected to *the “AWIN welfare assessment protocol for donkeys”* [57], performed by the same veterinarian, with specific training in animal welfare. The following points were evaluated:

- Body Condition Score
- Skin tent test
- Absence of injuries: integument alterations, swollen joints, lameness, prolapse
- Absence of disease (hair coat condition, faecal soiling, discharges, cheek palpation, abnormal breathing, coughing)

All donkeys included in this present study presented a 3/5 point-score, negative skin tent test and absence of injuries, and later the donkeys were divided into two groups: control groups (CG) and experimental groups (EG_1_ - EG_2_). Diurnal ethograms were compilated for all groups.

The control group (CG) was made up of 8 healthy Sardinian breed subjects: six females and two stallions, 6 ± 2.20 years old, housed in a milk production farm located in Ragusa (Italy), without any experience in AAIs. All animals were housed in a paddock of about 2000 m^2^ during the day and moved during the night to a paddock of about 800 m^2^ with a collective box of about 70 m^2^. The daily ration included a single distribution of hay (5.0 kg/animal/day) and commercial concentrate feed (2.5 kg/animal/day labelled to contain crude protein 13.00%, ether extract oil 3.20%, crude fiber 13.00%, ashes 10.50%, sodium 0.50%, lysine 0.44%, methionine 0.21%) and they were free to graze at all times, while water was available ad libitum. The data were collected from April to September 2018. 

The experimental group was divided into two subgroups: EG_1_ consisted of six Sardinian breed females, with an average age of 8 ± 3.7 years old, housed in the same farm, and subjected to the same management conditions of CG. They were used for AAT, and data recording was performed in the same period of CG. EG_2_ consisted of seven Amiata breed donkeys, with an average age of 6 ± 3.25 years (5 females and 2 stallions), housed in a rehabilitation center of a therapeutic community, located in Reggio Calabria (Italy), used for AAE. The daily ratio included a single distribution of hay (5.0 kg/animal/day) and commercial concentrate feed (2.5 kg/animal/day, labelled to contain crude protein 13.00%, ether extract 3.20%, crude fiber 13.00%, ashes 10.50%, sodium 0.50%, lysine 0.44%, methionine 0.21%), grazing on alternate days, and water was available ad libitum. The study was conducted during the period of April–August 2019. Donkeys were housed in a fenced paddock (800 m^2^), with a ground floor with a collective box (about 40 m^2^) located in a corner. All animal care was performed by the guests of the rehabilitation community.

In both areas of study, the environmental conditions followed the normal seasonal pattern for the location (15–30 °C; 20–60% RH—www.meteoblu.com).

EG_1_ patients were six male adults affected by personality disorders (F60–F69) and schizoid forms (F20), according to the tenth revision of the World Health Organization (WHO) international classification of diseases (ICD-10), ex ICD-9 code, diagnostic code 295.8, according to the classifier International Classification of Functioning, Disability and Health (ICF). During the work sessions, the therapist himself modulated the human–animal interaction.

EG_2_ donkeys carried out a working session with 4 adult subjects, 3 males, aged between 16 and 24 years old, guests of the therapeutic community and one male, 42 years old, a long-term care guest of the community, in a nosographic picture like that of EG_1_.

The donkeys’ full time diurnal ethogram (CG 05:00 a.m.–08:00 p.m.; EG_1_ 05:00 a.m.–07:00 p.m.; EG_2_ 05:00 a.m.–05:00 p.m.) was developed to select behavioral states or behavioral events according to the available scientific literature [35,38,39,57]. Table 1 shows the ethogram in which descriptions of behavioral states or behavioral events are reported. 

Continuous recording was performed by means of Hard Disk Drive (HDD) digital video camera systems by the same operator using the focal animal technique [58] for each observed group. The entire recording period (CG: 15 h; EG_1_: 14 h; EG_2_: 12 h) was examined in 30 min datasheets following the checklist of Table 2, used for quantified behavioral states and events of donkeys. The rows correspond to each 30 min of recording, and the columns correspond to every single state or behavioral event. The duration of each behavioral status was expressed in minutes; the frequency of each event was expressed in an arbitrary unit. The total value for the frequencies of behavioral events, in particular for play and vocalizations, was calculated. The different types of play and vocalizations may represent valid and highly critical indicators for reciprocal compliance between the donkey and the patient. We want to deepen the knowledge about this aspect.

### 2.2. Evaluation Test of the Donkey’s Behavioral Suitability for Use in AAI

The evaluation of animals’ temperament is a necessary condition to establish the suitability of animals used for AAIs. Whereas there is a well-known and widespread literature regarding temperament assessment tests on horses [50,59,60], only one study has been performed on donkeys by Gonzalez-De Cara et al. [26]. The correct animal selection during AAIs is fundamental to avoid any human patients’ danger. Furthermore, it is important not to cause distress for animals, according to the purposes and general target of the AAIs. For the evaluation and selection of suitable donkeys for AAIs, we used a part of the avoidance test proposed by AWIN [57] (starting position and testing phase), a novel object test, and an unknown person test [24] adapted for donkeys.

(A) Avoidance Test

(a1) Starting Position

The examiner was in front of the donkey that we wanted to assess. The distance between the examiner and the donkey was approximately 3.5 m, then he moved laterally (right or left) by 45° and observed the animal without moving his arms. If the donkey remained in attention position looking at him for 10–15 s. the observer moved his arm, and if the donkey did not take the avoidance position, the test was passed.

(a2) Testing Phase

The operator standing at the side of the animal (1.5–2.0 m away) approached it at the shoulder and gently placed a hand on it, once with the palm and once with the back of it, going through the withers, and observed the attitude of the auricles, and the position of the head and tail. If the animal did not show responses of avoidance, flight, or fear, the temperament test was passed.

(B) Novel Object Tests

We placed an unknown object (tripod) in the center of the collective habitual box and a known person invited the subject to enter and to achieve the center of it; if the subject followed without delay and did not stop, the test was passed.

(C) Unknown Person Test

A second evaluator, unknown to the donkey tested, entered into the collective box, where the donkey had previously been brought, remaining still; if the donkey approached the unknown person within 3 min, the test was passed; however, if the donkey stopped in the position and pointed to the evaluator, the test was negative.

The temperament evaluation test performed on 11 female subjects from the two EGs allowed us to select four subjects for each group, for their evaluation during the AAI sessions. 

### 2.3. Interaction Animal-Patient and Animal-Animal Monitoring

The AAI sessions lasting between 30 and 40 min were taped, and each of these consisted of the animal approach, presentation, contact (see avoidance test a1 and a2), hand control activity (brusque and curry), hand conduct (lead wire with a snap hook on the halter), detachment (grooming at the withers, vocal gratifications and removal).

(a) Interaction Animal-Patient monitoring
(b) Interaction Animal-Animal monitoring (social interaction)

(a) Interaction Animal-Patient monitoring

The four subjects of the EG_1_ and EG_2_ were used individually. The analysis of video setting sequences in a frame to frame through the compilation of an ethological check-list (Table 3 and Table 4) was done. The presence/absence of significant elements of the co-participation level and/or empathic involvement of the animal during the AAI sessions was evaluated [27], together with the detection of:

- olfactory/investigative interactions (smell, sniff)
- tactile donkey-patient tactics,
- the postural attitudes of the donkey

**Figure 1:**
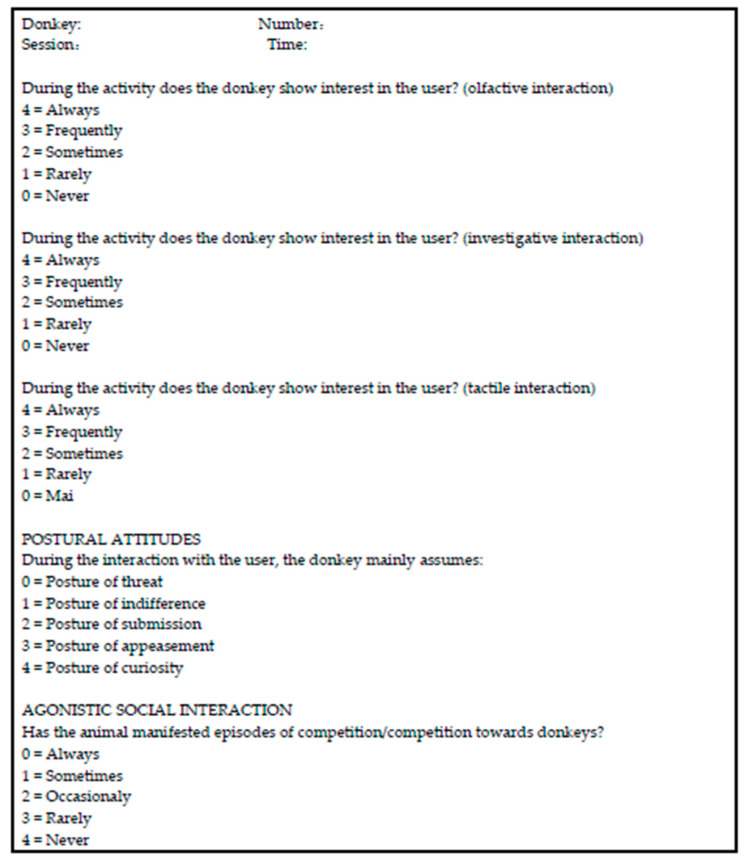
Observation form for the donkey’s behavior in during animal-assisted interventions (AAIs).

**Figure 2:**
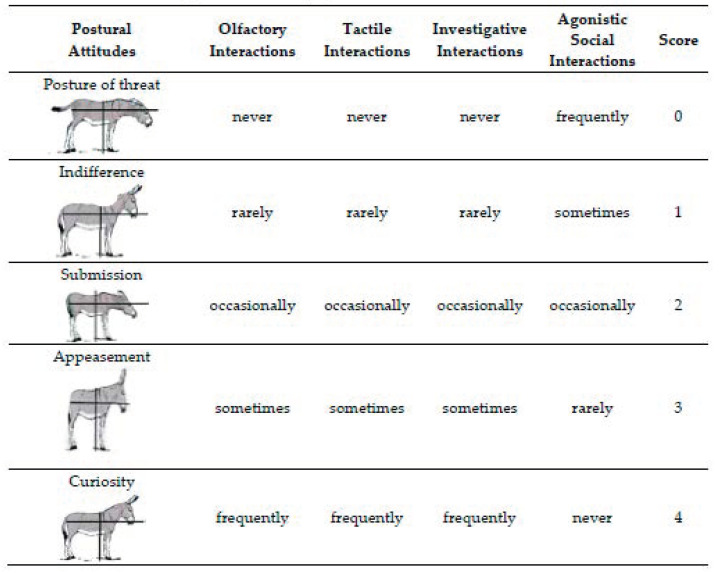
Qualitative judgment of binomial human-animal.

The expression level of each indicator was expressed in an arbitrary scale, with a range between a minimum of 0 (very low) and a maximum of 4 (very high), according to Figure 1 and Figure 2.

Based on the interpretation of the multiple postures, we assigned a score of 0 to the posture of threat, 1 to the posture of indifference, 2 to the posture of submission, 3 to the posture of appeasement, and 4 to the posture of curiosity [61]. The evaluation sheet of the ethological indicators was used for each subject during the individual sessions.

The attribution of value to the expected behavioral frameworks provided a preliminary evaluation of the animal’s suitability, according to the following scale:

- Total score between 0 and 10 = not suitable;
- Total score between 11 and 15 = to be used in controlled conditions;
- Total score between 16 and 20 = suitable.

(b) Interaction Animal-Animal Monitoring (Social Interaction)

Social interaction was evaluated by grooming; moreover, we observed other ethological indicators considered as an expression of animal welfare, such as nibbling self-grooming, rubbing self-grooming, bathing/rolling. All observations were performed when all donkeys were in the paddock (Table 3).

### 2.4. Heart Rate (HR) and Heart Rate Variability (rMSSD)

The HR values were detected using both a Polar S610i and Polar V800S Polar^®^ telemetric heart rate monitor for real-time recording, with scanning every 5 s, heart rate monitor activity (expressed in beats per minute, bpm). The Polar S610i heart rate monitor was placed with a health check band in the auscultation area of the cardiac ichthys, while the Polar V800S with Bluetooth technology was worn by the operator, and the polar belt equine chest strap was placed on the donkey. The obtained data were transferred, through an infrared or Bluetooth port, to a PC equipped with Polar Horse SW 4.0 or Polar FlowSync software Polar^®^ and edited graphically according to preset macros. The entire temporal session of the heart rate monitor data was transferred and edited on a spreadsheet for the processing of descriptive statistics (average, minimum, and maximum bpm values of each subject and group average values). In EG_1_ and EG_2_, HR was monitored during an AAI session; for 10 min before the session (T_0_); during the session for 10–20 min (T_1_), and only in EG_2_ for 10 min after the session (T_2_).

The data of the R-R interval were acquired using the Polar V800S (Polar^®^) for recording time of about 40 min (10’ T_0_ + 20’ T_1_ + 10’ T_2_) in each animal of the EG_2_. For the analysis of the cardiac variability signal, rMSSD obtained by the Polar FlowSync^®^ was considered as a statistical variable derived from the square root of the average of the sum of the squares of the differences between adjacent R-R inter-series. These data were assessed because through the trend of cardiac dynamics it is possible to acquire a series of quantitative and qualitative information to understand the level of activity of the ANS. The different types of heart rate time domain provide specific multiple valence indicators. The rMSSD estimates the activity of the parasympathetic system in a specific period of time. Low rMSSD values indicate a low parasympathetic tone, as in the case of situations with high emotional stress [62,63].

### 2.5. Data Analysis

Time budget of behavioral states or frequency behavioral events.

In the entire observation period, the duration of each behavioral state was obtained through an electronic spreadsheet prepared with command strings to link the subtotals of each datasheet. The time values of each behavioral state were rendered as a percentage (%) of the entire recording period set equal to 100.

### 2.6. Statistic

The Kolmogorov–Smirnov test was applied to verify the normal distribution of data (*p* > 0.05). Mann–Whitney-U tests for the comparison between not normally distributed data were applied to compare the HR values recorded in EG_1_. One-way analysis of variance (ANOVA) for repeated measurement was applied to the normally distributed data (HR and rMSSD values in EG_2_), using software STATISTICA 7.0 (Statsoft, Inc., Palo Alto, CA, USA).

### 2.7. Ethics Statement

Special permission for use of animals (donkeys) in this kind of behavioral study is not required in Italy. All procedures were performed in full accordance with Italian legal regulations (National Directive n. 26/14 – Directive 2010/63/UE) and the guidelines for the treatment of animals in behavioral research and teaching of the Association for the Study of Animal Behavior (ASAB). A consent to video-record and use data in an anonymous form was obtained by the parents and health personnel prior to study.

## 3. Results

### 3.1. Ethograms

(a) The Diurnal Ethogram (05:00 a.m.–08:00 p.m.) of the CG

**Figure 3:**
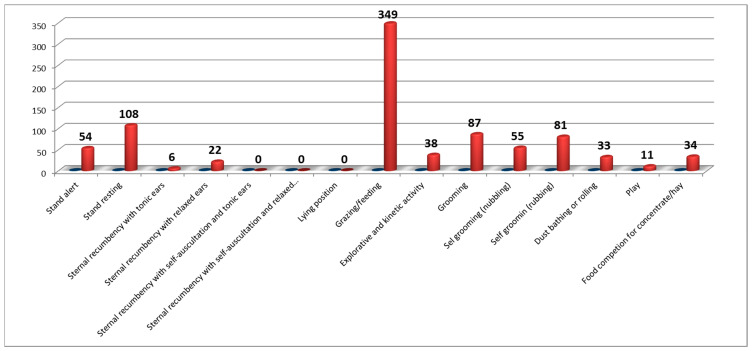
Diurnal ethogram (05:00 a.m.–08:00 p.m.) of the CG donkeys.

Figure 3 shows the clear prevalence of grazing and feeding (349 min; 38% of total activity) compared to all other behavioral categories. The prevalence of the posture in stand resting (108 min; 12%) emerged compared to that in stand alert (54 min; 6%), exploratory and kinetic activities (38 min; 4%), and play activity (11 min; 1%). The behavioral categories that arouse speculative interest are represented by the duration of the grooming activities (87 min; 10%) and self-grooming (136 min; 15%) and the levels of activity of the rolling or dust bathing (33 min; 4%) as an indicator of animal welfare.

(b) The Diurnal Ethogram of the EG_1_ (05:00 a.m.–07:00 p.m.) and EG_2_ (05:00 a.m.–05:00 p.m.)

**Figure 4:**
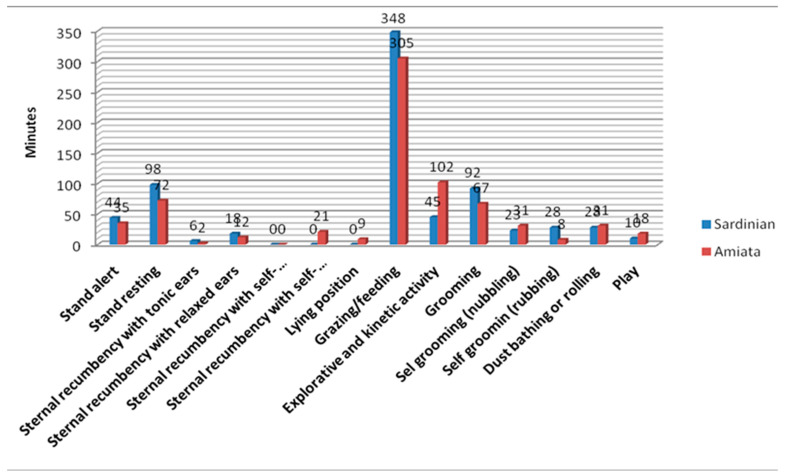
Diurnal ethogram of the EG_1_ (05:00 a.m.–07:00 p.m.) and EG_2_ (05:00 a.m.–05:00 p.m.) donkeys.

The ethograms study of the EG_1_ and EG_2_ confirmed the clear prevalence of grazing and feeding of the Sardinian breed of donkey, as well as the Amiata donkey, compared to all the other behavioral categories (Figure 4). Comparing the diurnal ethograms of EG_1_ and EG_2_ vs. CG, it is possible to highlight the suitability of the behavioral manifestations levels of activity connected with sociability, gregariousness, and peer attachment. 

In CG, grooming and play comprised 12% of daytime behavioral activities. In EG_1_ and EG_2_, the prevalence of these activities was 13% and 11%, respectively.

These behavioral expressions showed suitable welfare conditions and therefore the suitability of the subjects used for the AAIs.

### 3.2. Heart Rate (HR) and Heart Rate Variability (HRV) EG_1_ and EG_2_

The mean HR values in EG_1_-T_1_ were statistically significantly different (70.40 ± 7.60) (*p* < 0.05), compared to T_0_ HR mean values (58.60 ± 2.0) (Table 4).

Mean HR values of EG_2_ donkeys are reported in Table 5.

**Figure 5:**
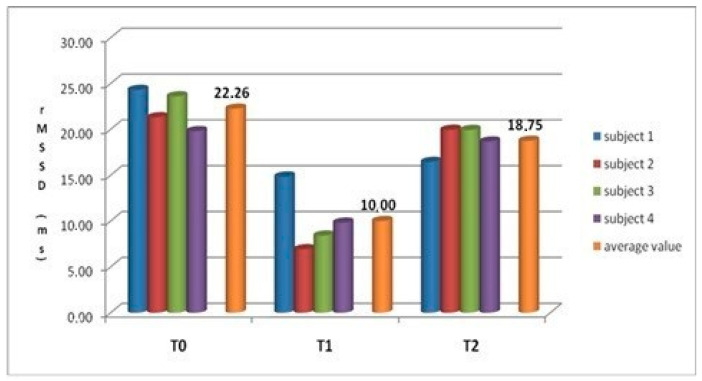
Individual and average values of rMSSD of Amiata donkeys EG_2_ group at the times T_0_, T_1,_ and T_2_ of the AAIs session.

Mean values of rMSSD recorded in EG_2_ showed statistically significant differences at the time T_1_ vs. T_0_, calculated by an ANOVA test of repeated measurements (*p* < 0.05) (Table 6 and Figure 5).

We believe that the obtained data regarding the rMSSD values in the EG_2_ are extremely interesting. The parasympathetic tone prevailed over the sympathetic tone at the T_0_, the rMSSD showed the highest value, indicating low or absent stress. In the EG_2_-T_1_ during the AAIs session, HR showed the highest value, and the rMSSD showed the lowest value, confirming the activity of the sympathetic tone and the conditions of emotional discomfort.

In the post session, the rMSSD average values were 18.75 ms, although not statistically substantially different from the basal ones (22.26 ms), which demonstrated that the refreshment times must be commensurate with the nosographic pictures of patients. It must be individualized to the donkey subject and, last but not least, cannot be understood only as no working time but must have connotations of emotional refreshment that is possible only in large paddocks and with an adequate period of time.

### 3.3. The Human-Animal Interactions Evaluation

All EG_1_ and EG_2_ subjects scored in the range 16–20 (see Table 3 and Table 4), and therefore are suitable for AAIs activities, as shown in Table 7. In particular, in the EG_2_ group, subjects No. 1 and 3 proved to be particularly suitable for use in AAIs.

## 4. Discussions

Our results show that it is essential to fill in the ethogram of animals before evaluating the effects of the AAI approach on animal welfare. In fact, only after verifying that the management conditions have not changed the ethological and physiological indicators of welfare, it is possible monitoring any changes induced by the IAA sessions.

Nowadays, in most of the structures that carry out AAIs, the organization of management, refreshment times, and the activities to support animals’ ethological needs are often overlooked. Consequently, it is possible to believe that animal welfare is not duly considered, invalidating the purpose of the relational and/or therapeutic intervention. Frequently, we are forced to add that the beneficial effects deriving from a correct donkey–patient “dialogue” do not correspond to the appropriate animal welfare protections, betraying the spirit of therapeutic intervention. In particular, the obtained results about the preliminary assessment of the donkey’s living environment quality represent a fundamental aspect of the therapeutic projects. The preliminary compilation of the diurnal ethogram of donkeys used for the AAIs can highlight specific behavioral deprivations.

Grooming activity and the various types of self-grooming are important indicators of the ethological evaluation of sociability, peer attachment, and good welfare.

The types of management and the semi-stable conditions of the studied donkeys allowed us to highlight unsuitable living spaces for the expression of various behavioral manifestations linked to the animal’s emotional integrity, such as exploration, standing alert, and playing.

The evaluation of the quality of animals’ life used for AAIs should be done by applying *“Guidance on Risk Assessment for Animal Welfare; EFSA Panel on Animal Health and Welfare* “ [64] and the Animal Welfare Index for donkey [57].

The Sicilian Region authorization procedure [65] for the structures that provide AAI services (Table 8.) establishes that the individual donkey boxes must meet the minimum measures for donkeys reported by AWIN protocol for this species [57] and adopted by the Swiss Animal Welfare Ordinance 23 April 2008 [66] for a collective boxes (Table 9.).

Meanwhile, the collective boxes must have the following minimum measurements: 

The planning of the AATs and AAEs must provide a reasonable emotional refreshment time of the animals between a session and the next or, at the end of the activities, as the HR and rMSSD values detected at time T2 showed.

In order to mitigate any stress induced by the AAT/AAE, it is appropriate to allow donkeys to perform free movement in groups, for the reaching of so-called social buffering, to guarantee strong sociability and gregariousness of the donkeys, Therefore, it is recommended that donkeys are housed in collective shelters, taking care to verify social affinity first.

The ethological evaluation models according to the obtained results fill the methodological gaps currently present in the AAI scenario, providing a tool of undebatable efficacy to protect animal welfare and desired therapeutic efficacy of caring relationship. With reference to the third part of our research project, the neurovegetative indicators (HR and rMSSD) of the emotional involvement of donkeys used for AAIs represent a starting point for the definition of a standardization protocol for donkeys used in AAIs.

At least, regarding the statistically significant differences in the average HR values in the subjects of the EG, and in the average rMSSD values, particular attention should be paid to the duration of the AAIs sessions and, above all, to the number of sessions with the same subject, providing for a suitable refreshment time between one session and another, represented by the return to the social group and suitable spaces for self-grooming activities (dust bathing). 

Moreover, in the HRV analysis, it is of special interest to evaluate the power and the frequency of the signal within certain pre-defined frequency bands [67]. VLF bands represent the variations in the frequency which are influenced by regulatory mechanisms, such as the renin–angiotensin system and thermoregulation; LF represents the variation that is associated to the orthosympathetic/parasympathetic modulation; and HF represents variations in frequency secondary to the respiration, and is mediated by the parasympathetic nervous system [67]. HRV has been measured in different species, including horses. It has been used to monitor the response to mental stress in association with cortisol [68,69] or with HR and selected behavioral parameters [22]. It is also useful to evaluate the response to challenging objects [70] or to assess pain associated with laminitis [71]. To conclude, HRV analysis in animals used in AAIs appears to be a sensitive measure of both physical and emotional stress responses. We want to deepen the knowledge about the frequency spectrum analysis of HRV during AAT and AAE session in donkeys.

The benefits achievable through the human–animal relationship have been largely investigated for several categories of patients, such as, for example, children with autism spectrum disorder [4,72,73], elderly patients affected by dementia, or psychiatric disorders [74,75,76], and alcohol/drug-addicted inmates [77,78,79], even though a need for more evidence-based research still persists [80].

Scientific studies on AAT, particularly those involving dogs, have shown an openness to and acceptance of this strategy by medical teams [81,82,83,84,85,86,87] and have documented recognition for AAT’s safety and efficacy in different environments and clinical contexts, including hospitalization [88,89,90], emergency medicine [86], oncology [91,92,93,94,95,96,97], cardiology [87,98], psychiatry [99,100,101], and outpatient and hospital pediatrics [102,103,104,105,106].

The results of studies on AAT are promising despite the lack of standardization of the number, duration, and frequency of sessions, the executed activities, and the safety measures for the animals and patients.

From the social point of view, the growing interest in the topic by families or disabled people and therapist and educators has raised the necessity to regulate and structure the sector, taking into account many issues about the involvement of animals in activities related to human health and wellbeing and stressing ethical [107], safety [108] and economic arguments [109]. Hence, at the international level, some associations and organizations have developed and established standards and best practices for AAI, as in the case of the White Paper of the International Association of Human-Animal Interaction Organizations [18], or the Animal-Assisted Interventions Code of Practice for the UK, edited by the Society for Companion Animal Studies [19].

Considering that the assigned subjects to the AAIs setting had been selected by temperament and that the social level checklist proved the suitability of the relationship, we must take into account, first of all, the risk for the operator and the patient to work with unsuitable subjects and, last but not least, the demonstrated stress that the AAI session causes to the donkey.

## 5. Conclusions

On the basis of the scientific evidence discussed in this article, it is possible to highlight the fundamental elements for the definition of a protocol to evaluate the donkey’s behavioral suitability to be used for AAIs, as a tool to support the judgment on the basis of the set of factors–parameters: management, experiential level, the setting of employment risks and type and level of relationship with the patient. Procedurally, the suitable and treated elements to outline a donkey’s ability to be used for AAIs can be identified in the evaluation of the level of reactivity/sociability, which, together with the results of the test for the evaluation of donkey’s welfare and the management conditions, is established by the presence of specific infrastructure and management requirements, as previously treated, which are suitable elements for the responsible use of donkeys in AAIs.

## Figures and Tables

**Table 1:** Ethogram: description of the selected behavioral states or behavioral events of *Equus asinus L.*

stand alert	Quadrupedal or tripodal position, angle formed by the straight line tangent to the top edge of the neck > o = 45°, auricles erect and in active orientation
stand resting	Quadrupedal or tripodal position, angle formed by the straight line tangent to the top edge of the neck < or = 0°, with tonic or relaxed ears
Resting: sternal recumbency with tonic or relaxed ears	Sternal recumbency: rest or sleep while lying down with its head up or with its legs and head stretched out, with tonic or relaxed ear
Resting: “self auscultation posture, with tonic or relaxed ears”	Lying down on sternum, legs folded underneath body frame
Lying position	Lateral recumbency: rest or sleep while lying down with its head up or with its legs and head stretched out, with tonic or relaxed ears
Grazing and feeding	head extended on the neck, gluing stretched towards the ground in the act of drawing from the substrate, often accompanied by slow and very discontinuous walking; the use of wall feeders has altered the most typical attitude of this behavior as well the supplementing food with concentrate/hay
Explorative and kinetic activities explorative/kinetic	progression in space through the coordination of the limbs more or less fast
Mutual grooming	motor coordination that allows a subject to groom the skin surface of a similar; more frequently through the neck, withers, and rump regions
Self-grooming (nubbling, rubbing)	motor coordination that allows a subject to groom its body surface, through a limb or nibbling (nibbling, rubbing)
Dustbathing or rolling	Characteristic fixed-action pattern (FAP) of the donkey used for self-grooming. The subject incomplete lateral recumbency rotates on its longitudinal axis alternately to the right and left, giving it the push with the contraction of the lumbar muscles and the neck, extending the head. The dust bathing has a significant value between the behavior of maintaining emotional homeostasis This performance has the relaxation meaning or emotional tension discharge.
Play	motor coordination: wheelies, buckings, nibbles, incarcerations with the gluing of the like, galloped in a circle or a straight line
Food competition for concentrate/hay	Turn towards consisted of simple bending of the neck so that the sender was looking more directly at the receiver. Fight: two adult subjects threaten each other with ears back and/or mouth open and head horizontal; walk chase directed at another and head down;
Vocalization	Bray, grunt, growl, whuffle, snort
Drink	head extended on the neck with gluing stretched in the act of drawing from the water source; the use of automatic drinkers has altered the most typical attitude of this behavior

**Table 2:** Checklist used to quantify behavioral states or behavioral events of *Equus asinus L.*

**Time Laps (min)**	stand alert	stand resting	sternal recumbency with tonic ears	sternal recumbency with relaxed ears	“self - auscultation posture”: lateral recumbency with tonic ears	“self-auscultation posture”: lateral recumbency with relaxed ears;	lying position	grazing and feeding	explorative and kinetic activities explorative/kinetic	mutual grooming	self-grooming (nubbling)	Self-grooming (rubbing)	dustbathing or rolling	play	food competition for concentrate/hay	vocalization	drink
1																	
2																	
3																	
4																	
5																	
6																	
**Up to 30**																	

**Table 3:** Monitoring of donkey ethological indicators of welfare.

Subject	Grooming	Self-Grooming (Nibbling)	Dustbathing or Rolling	Self-Grooming (Rubbing)
1				
2				
3				
......				
Total				

**Table 4:** Average values (Mean ± S.D.) of the heart rate, expressed in beats/minute (bpm) of Sardinian donkeys (experimental group EG_1_).

Heart Rate Monitoring Group EG_1_	
Group (n. 4 subjects)	Average Values of Heart Rate (bpm) M ± S.D.
EG_1_-T_0_	58.60 ± 2.00
EG_1_-T_1_	70.40 ± 7.60 *

* *p* < 0.05.

**Table 5:** Average values (Mean ± S.D.) of the heart rate—expressed in beats/minute (bpm)—of Amiata donkeys (experimental group EG_2_), at the times T_0_, T_1,_ and T_2_ of the AAI session.

Heart Rate Monitoring Group EG_2_	
Group (n. 4 Subjects)	Average Values (bpm) M ± S.D.
EG_2_-T_0_ (10 min)	66.5 ± 4.18
EG_2_-T_1_ (15–20 min)	90.00 ± 6.34 *
EG_2_-T_2_ (10 min)	74.12 ± 2.02

* ANOVA test: EG_2_-T_1_ vs. EG_2_-T_0_; *p* < 0.001.

**Table 6:** Average values (M ± S.D.) of the rMSSD—expressed in ms—of Amiata donkeys (experimental group EG_2_) at the times T_0_, T_1,_ and T_2_ of the AAIs session.

rMSSD EG_2_ Group	
Group (n. 4 Subjects)	Average Values (ms) M ± S.D.
EG_2_-T_0_ (10 min)	22.26 ± 2.07
EG_2_-T_1_ (15–20 min)	10.00 ± 3.44 *
EG_2_-T_2_ (10 min)	18.75 ± 1.65

* Test ANOVA with repeated measurements: EG_2_-T_0_ vs. EG_2_-T_1_, *p* < 0.05.

**Table 7:** Behavioral scores of each donkey in the EG_1_ and EG_2_ group after observation with patient code F60–F69, F20 (ICD10).

Subjects.EG_1_	Postural Attitude		Olfactory Interaction		Tactile Interaction		Investigative Interaction		Agonistic Social Interaction		Total Score	
	EG_1_	EG_2_	EG_1_	EG_2_	EG_1_	EG_2_	EG_1_	EG_2_	EG_1_	EG_2_	EG_1_	EG_2_
1	3	4	3	4	3	4	4	4	3	4	**16**	**20**
2	4	3	3	3	3	3	3	3	4	4	**17**	**16**
3	4	4	3	4	4	4	4	4	4	4	**19**	**20**
4	3	3	4	4	4	4	3	4	4	3	**18**	**18**
**Total**	**14**	**14**	**13**	**15**	**14**	**15**	**14**	**15**	**17**	**15**		

**Table 8:** Minimum measurements for individual boxes.

Withers	<120 cm	120–148 cm	>148–162 cm	>162–175 cm
Minimum area (in m^2^)	5.5	7.0	8.0	9.0
Minimum width of the box (in m)	At least one and a half times that of the withers	At least one and a half times that of the withers	At least one and a half times that of the withers	At least one and a half times that of the withers

by Health Department of Sicilian Region [65].

**Table 9:** Minimum measurements for collective boxes.

Withers	<120 cm	120–134 cm	>134–148 cm	>148–162 cm	>162–175 cm	>175 cm
Minimum area for equid (in m^2^)	5.5	7.0	8.0	9.0	10.5	12.0

by Health Department of Sicilian Region [65].
